# Effectiveness of physical exercise on mental health among university students: a systematic review and meta-analysis

**DOI:** 10.3389/fpsyg.2025.1612408

**Published:** 2025-11-19

**Authors:** Longyan Liu, Xianyang Xin, Hai Wang, Ying Zhang

**Affiliations:** College of Physical Education and Training, Capital University of Physical Education and Sports, Beijing, China

**Keywords:** physical activity, mental health, university students, meta-analysis, RCT

## Abstract

**Introduction:**

The global incidence of adverse mental health symptoms among university students at various stages has been increasing year by year. Compared to passive medical treatments, physical exercise, as a rehabilitative therapeutic approach, appears to play a significant role in preventing poor mental health among students. This meta-analysis aims to evaluate the impact of physical activity interventions on the mental health status of university students.

**Method:**

This study systematically searched PubMed, Cochrane Library, Web of Science, Embase, and Scopus for randomized controlled trials (RCTs) from the inception of the databases up to March 2025. The study participants were university students, and the outcome measures included mental health indicators such as wellbeing, anxiety, depression, stress, and sleep quality. Meta-analysis of the included studies was conducted using Review Manager 5.4 and Stata 16 software.

**Results:**

A total of 34 articles comprising 80 individual studies and 8,020 participants were included in the meta-analysis. Physical activity interventions were significantly associated with improvements in university students’ mental health outcomes. Specifically, exercise demonstrated a large positive effect on overall mental health (SMD = 0.91, 95% CI: 0.15 to 1.67), enhanced wellbeing (SMD = 0.41, 95% CI: 0.29 to 0.53), and led to moderate reductions in anxiety (SMD = −0.62, 95% CI: −0.84 to −0.41), depression (SMD = −0.67, 95% CI: −0.91 to −0.43), and stress (SMD = −0.46, 95% CI: −0.64 to −0.28). Sleep quality also improved significantly (SMD = −0.57, 95% CI: −0.74 to −0.40). Subgroup analyses indicated that interventions with a lower frequency (≤3 sessions per week) and longer duration (10–48 weeks) were particularly effective. Among exercise types, resistance training and high-intensity interval training showed the strongest effects on anxiety, depression, and stress reduction, while aerobic exercise was most effective for improving sleep quality.

**Conclusion:**

Active participation in physical exercise has been shown to significantly improve the mental health of university students. To further enhance students’ psychological wellbeing and prevent mental health disorders, it is crucial to promote regular physical activity. Engaging in regular exercise can help alleviate symptoms of depression, anxiety, and stress, while also improving overall wellbeing and sleep quality.

**Systematic review registration:**

PROSPERO registration: CRD420251016260; publicly accessible at: https://www.crd.york.ac.uk/prospero/.

## 1 Introduction

Over the past two decades, mental health issues among university students have garnered significant attention from society and emerged as a prominent topic within the field of psychology (Lin et al., 2022). Students face numerous pressures, including academic challenges, the constant drive to succeed, competition with peers, limited leisure time, and reduced opportunities for family interactions. Additionally, they frequently worry about the future, and in some underdeveloped countries or regions, they also confront significant financial difficulties (Mikolajczyk et al., 2008; Tosevski et al., 2010). Consequently, university students are more prone to experiencing various mental health issues (Gore et al., 2011). For instance, a global study conducted in 2017 assessed 13,984 university students and revealed that 35% of them exhibited at least one positive indicator of a mental disorder (Auerbach et al., 2018). Moreover, over the past decade, the prevalence of depression and anxiety symptoms among university students has steadily increased (Lattie et al., 2019). Globally, approximately 31% of university students have undergone screening for mental health disorders (Auerbach et al., 2018). A survey conducted in the United States involving 14,175 university students found that the prevalence of depression was 17.3%, anxiety was 7.0%, suicidal ideation was 6.3%, and non-suicidal self-injury was 15.3% (Eisenberg et al., 2013). The World Health Organization’s “World Mental Health International College Student (WMH-ICS) Initiative” revealed that, among the eight countries surveyed, 31.4% of first-year students had been screened for at least one mental health issue in the past year (Pei et al., 2024). Chen et al. (2022) found that the detection rates of anxiety, depression, sleep disorders, and suicide attempts among university students had significantly increased. The detection rate of anxiety was 13.7%, depression was 20.8%, and sleep problems were 23.5%, all highlighting serious mental health concerns among students. Students are regarded as the nation’s capital and the primary investment for the future, as they constitute the key group determining a country’s economic growth and success (Auerbach et al., 2018). Therefore, it is essential to understand and address the mental health issues faced by university students.

Due to the low level of mental health literacy among many students, they often fail to recognize the need for treatment and instead perceive symptoms of depression and anxiety as typical university stress, believing that no intervention is required (Eisenberg et al., 2007). Even students who acknowledge the need for mental health services often face numerous barriers when seeking care, including financial constraints, negative attitudes and perceptions toward mental health services (including stigma), concerns about privacy, and lack of time (Eisenberg et al., 2011). For instance, Eisenberg et al. (2007) found that among students diagnosed with severe depression, only 36% had received medication or psychotherapy in the past year. When university students experience mental health issues, they often attempt to resolve them independently, with professional counseling or medication being considered a last resort only when other approaches fail (Fortney et al., 2016). According to previous studies, the primary treatments for anxiety disorders currently include medication with serotonin reuptake inhibitors and cognitive behavioral therapy (CBT) (Taylor et al., 2012), with medication being the most commonly used treatment method (Lin and Gao, 2023). Although the effectiveness of medication in treating anxiety disorders has been confirmed, its side effects can be significant. There is also a risk of relapse after discontinuing medication, making it a potentially inadequate long-term solution for some patients (Broocks et al., 1998).

Participating in physical activities has been shown to be an effective strategy for improving physical health (Benaich et al., 2021) and preventing mental health issues (San Román-Mata et al., 2020). Additionally, some scholars have demonstrated that physical activities lead to various physiological changes that can enhance mood and reduce stress and anxiety levels (Carter et al., 2021). For example, regular physical activity is associated with increased self-confidence and lower levels of anxiety and depression. Furthermore, engaging in at least 150 min of moderate to vigorous physical activity (PA) per week can effectively prevent depressive symptoms (Peluso and Andrade, 2005), reduce anxiety and stress, and improve quality of life (San Román-Mata et al., 2020). As a result, participation in physical activities is increasingly recognized as an effective way to address mental health issues among university students. Moreover, studies have shown significant differences in mental health between students in sports-related majors and those in traditional liberal arts majors (Li and Shi, 2021), with students in sports majors exhibiting much better mental health than their peers in other fields (Frank et al., 2021). However, despite existing evidence indicating that physical activity (PA) can improve the mental health of undergraduate students (Melnyk et al., 2014; Herbert et al., 2020), some studies have reported inconsistent results. For example, Herbert et al. (2020) found that a six-week aerobic exercise intervention, compared to a waitlist control group, alleviated symptoms of depression and stress over time but had no significant effect on anxiety. In contrast, Ghorbani et al. (2014) conducted a randomized controlled trial and found that a six-week aerobic exercise program not only alleviated depression and sleep disorders among university students but also effectively reduced anxiety. Moreover, different levels of exercise intensity or variations in experimental design can have varying effects on improving mental health issues. For instance, Pascoe et al. (2014) found that physical activity interventions appeared to effectively reduce certain symptoms of poor mental health, particularly when the interventions involved higher-intensity physical activities. However, Arent et al. (2005) found that high-intensity resistance training might be associated with an increase in state anxiety. Given the rising incidence of mental health issues among university students and the crucial role this group plays in a nation’s development, this review aims to evaluate the effectiveness of physical activity participation on undergraduate students’ mental health outcomes. The goal is to better promote the development of physical education programs in universities and more effectively address mental health issues among university students.

## 2 Methods

This article presents a systematic review of randomized controlled trials (RCTs), following the Preferred Reporting Items for Systematic Reviews and Meta-Analyses (PRISMA) guidelines. Prior to screening the search results, the research adhered to the PRISMA statement. The protocol has been prospectively registered with the International Prospective Register of Systematic Reviews, with the registration number CRD420251016260, and complies with the PRISMA guidelines for systematic reviews and meta-analyses (Page et al., 2020)^1^.

### 2.1 Study selection

In the preliminary screening phase, all retrieved literature was imported into the reference management software EndNote X9 to remove duplicate records. Two researchers (LY and XY) then independently screened the titles and abstracts of the literature to identify all potentially relevant studies and supplemented missing information by contacting the authors. Studies that met the inclusion criteria were independently identified and evaluated by the same two researchers (LY and XY). Any disagreements were resolved through discussion, and if necessary, a third expert (YZ) was consulted.

The inclusion criteria were developed based on the Participants, Intervention, Comparison, Outcomes, and Study Design (PICOS) principles. The inclusion criteria were as follows: (1) Participants were enrolled in undergraduate university courses; (2) The physical activity intervention lasted for at least 4 weeks and was part of an experimental randomized controlled trial (RCT); (3)The study included a control group, such as a waitlist control, no treatment control, other health behavior comparison, or no control (e.g., single-arm or pre-post studies); (4) Outcome measures included psychological health status before and after the exercise intervention, such as wellbeing, anxiety, stress, depression, and sleep quality. The exclusion criteria were: (1) Non-English publications; (2) Dissertations, conference proceedings, or abstracts where the full text could not be obtained; (3) Studies from which valid outcome data could not be extracted, and contacting the authors was unsuccessful; (4) Duplicated publications.

### 2.2 Search strategy

The literature search included multiple databases: PubMed, Scopus, Cochrane Library, Web of Science, and Embase, with the search period covering database inception through March 2025. To ensure that relevant studies were not missed, we also reviewed the reference lists of systematic reviews published in the past 3 years. A search strategy combining subject headings and free terms was used, with medical subject headings (MeSH) applied as search terms for mental health outcomes where appropriate, along with keywords representing various types of physical exercise and ways to describe undergraduate students. To ensure no relevant studies were omitted, we also manually reviewed the reference lists of the included studies and conducted a grey literature search. The keywords included “exercise,” “undergraduate,” "physical activity,” "mental health,” "mental disorders,” "depression,” "anxiety,” “stress,” and “sleep disorders” to ensure comprehensive coverage.

### 2.3 Quality assessment

The risk of bias in the included studies was assessed by three researchers (L.Y., X.Y., and H.W.) using the Cochrane Handbook’s risk of bias tool for RCTs (Higgins et al., 2011). The focus was on evaluating risks related to random sequence generation, allocation concealment, blinding of participants and researchers, blinding of outcome assessment, incomplete outcome data, selective reporting, and other potential biases. Each factor was thoroughly assessed and classified as high risk, low risk, or unclear. This rigorous risk assessment process ensured the credibility of the studies and upheld the scientific integrity of the results. The three authors independently conducted the quality assessment, and any discrepancies were resolved by a four reviewer (Y.Z.).

### 2.4 Data extraction

Data extraction from the literature was conducted independently and in a double-blind manner by researchers. The extracted data included the first author, publication year, intervention details (sample size of each group, gender, age), intervention measures (type of exercise, duration, frequency, and exercise time), and outcome measures (pre- and post-test values for both the experimental and control groups). If outcome measures were not available in the full text, the corresponding author was contacted to request the data. If the data could not be obtained, the study was excluded.

### 2.5 Statistical analysis

Statistical analysis was conducted using Review Manager 5.4 and Stata 16 software for effect size pooling, subgroup analysis, sensitivity analysis, and publication bias tests. To minimize the impact of baseline differences, the change values of pre- and post-intervention means and standard deviations were used for effect size pooling. Since the included studies involved continuous variables with inconsistent measurement units, the effect sizes for outcome measures were represented using the Standardized Mean Difference (SMD) and its 95% confidence interval (95% CI). The statistical significance of pooled estimates was tested using *p*-values, with *p* ≤ 0.05 indicating statistical significance. Heterogeneity was assessed using I^2^, with low heterogeneity defined as *I*^2^ < 50%, moderate heterogeneity as 50% ≤ *I*^2^ < 75%, and high heterogeneity as *I*^2^ ≥ 75%. Based on the degree of heterogeneity, either a fixed-effects model or a random-effects model was used for the analysis of the included studies. When *I*^2^ ≥ 50%, sensitivity analysis was performed to test the stability of the results, and subgroup analysis was conducted based on study characteristics to explore the sources of heterogeneity. Forest plots were used to represent the statistical significance of the pooled effect size (SMD), with small effect size defined as SMD < 0.5, medium effect size as 0.5 ≤ SMD < 0.8, and large effect size as SMD ≥ 0.8. Publication bias in the included studies was quantitatively and qualitatively analyzed using the Berg test, Egger’s test, and funnel plots.

## 3 Results

### 3.1 Literature search results

**Figure 1:**
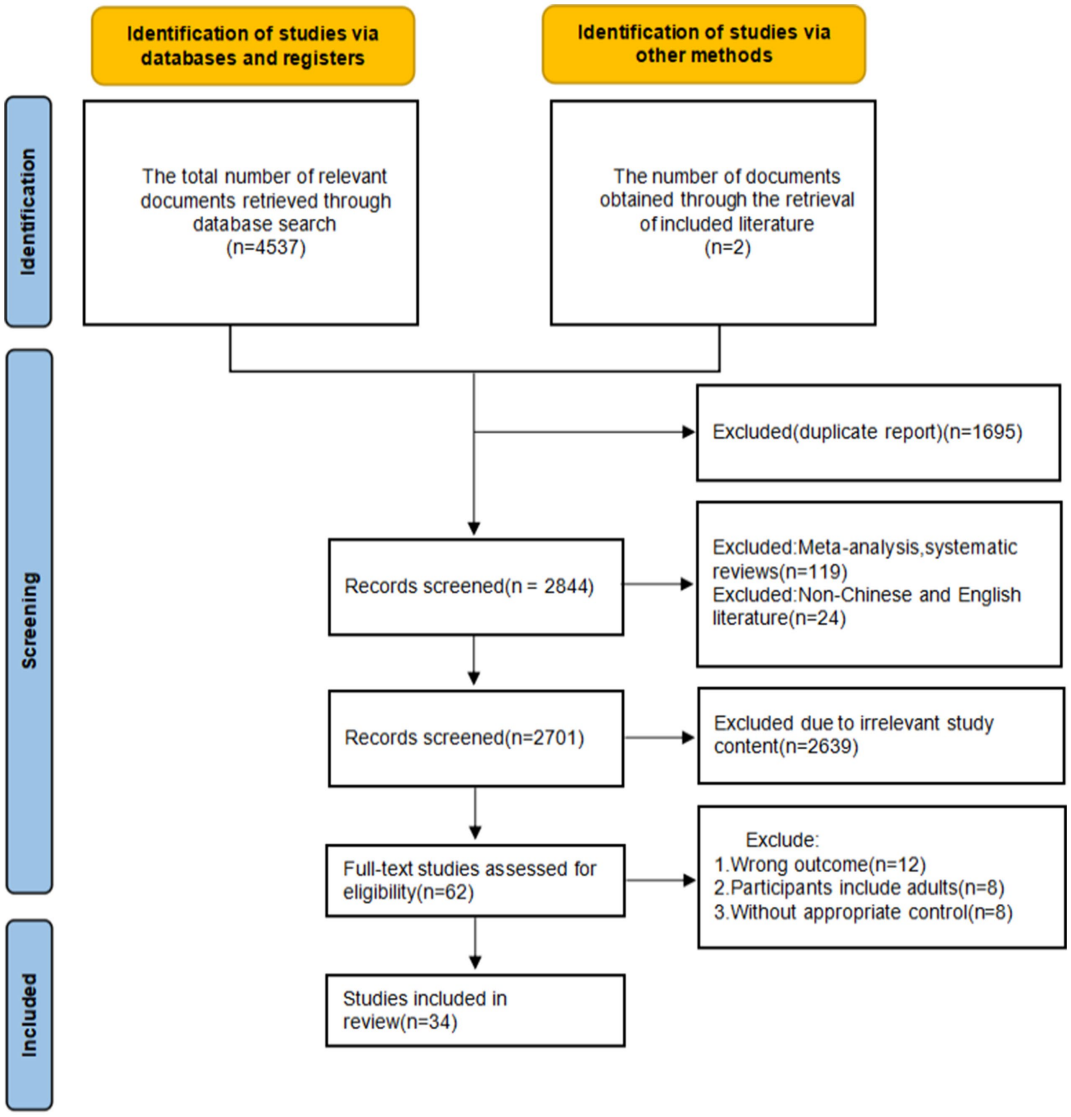
Flow chart of literature screening.

A total of 4,537 relevant articles were retrieved through database searches: PubMed (*n* = 1,165), Embase (*n* = 933), Cochrane Library (*n* = 839), Web of Science (*n* = 749), and Scopus (*n* = 851), with an additional 2 articles obtained through other sources. Duplicate articles (*n* = 1,695) were removed using EndNote 20 software. Two researchers independently screened the titles and abstracts, removing 2,639 articles, and further excluded 28 articles after full-text review. Ultimately, 34 articles were included. The literature selection process is illustrated in Figure 1.

### 3.2 Basic characteristics of included studies

A total of 34 articles were included, with publication years concentrated within the past 20 years, involving 8,020 participants (Ghorbani et al., 2014; Hurdiel et al., 2017; Chang et al., 2022; Antony and Tomar, 2019; Arbinaga et al., 2018; Aşçı, 2003; Brandão et al., 2024; Castellote-Caballero et al., 2024; De Vries et al., 2016; Erdoğan et al., 2024; Falsafi, 2016; Hemat-Far et al., 2012; Li K. et al., 2022; Lin Latt et al., 2024; Philippot et al., 2022; Saltan and Ankaralı, 2021; Shavaisi et al., 2024; Wang et al., 2024; Xiao et al., 2021; Zhang et al., 2023; Zhang and Jiang, 2023; Abavisani et al., 2019; Choi et al., 2018; De Vries et al., 2018; Eather et al., 2019; Ji et al., 2022; Kim, 2014; Li et al., 2015; Li R. et al., 2022; Paolucci et al., 2018; Tomar et al., 2023; Yiğiter and Hardee, 2017; Barği, 2022; Fukui et al., 2021). These articles encompassed 80 studies, as multiple research methods reported within a single article were counted as separate studies. Among these, the highest number focused on anxiety (24 studies), followed by stress (19 studies), depression (18 studies), sleep disorders (8 studies), wellbeing (6 studies), and general mental health (5 studies). The types of training interventions included aerobic exercise, resistance training, high-intensity interval training, and combined exercise. The basic characteristics of the included studies are presented in Tables 1, 2.

**Table 1:** Basic characteristics of the included studies.

Author	country	Research type	Participants (M/F)	Sample Size (N)		Age(Years)		Intervention characteristics	Intervention Duration (Weeks)	Session Duration	Session Frequency (times/week)
				Exercise	Control	Exercise	Control				
Antony and Tomar (2019)	Saudi Arabia	RCT	NR	20	20	18.52 ± 0.599	18.52 ± 0.599	AE	16	45 min	2
Arbinaga et al. (2018)	Spain	RCT	4/24	15	13	19.79 ± 1.71	19.93 ± 1.84	AE	7	70 min	3
Aşçı (2003)	Turkey	RCT	0/40	20	20	21.35 ± 0.88	21.20 ± 1.67	AE	10	50 min	3
Brandão et al. (2024)	Portugal	RCT	8/64	28	44	21.46 ± 5.70	20.52 ± 3.75	Yoga	6	60 min	6
Castellote-Caballero et al. (2024)	Spain	RCT	66/63	65	64	20.29 ± 1.78	20.30 ± 1.76	Yoga	12	60 min	2
Chang et al. (2022)	USA	RCT	NR	327	352	21.4 ± 4.7	21.3 ± 5.0	Yoga	4	25 min	7
De Vries et al. (2016)	Netherlands	RCT	19/80	50	49	20.9 ± 2.5	20.7 ± 2.2	AE	6	60 min	3
Erdoğan et al. (2024)	Turkey	RCT	0/80	39	40	20.56 ± 1.72	20.56 ± 1.72	Yoga	8	42 min	2
Falsafi (2016)	USA	RCT	8/52	30	30	20.29 ± 1.78	20.30 ± 1.76	Yoga	8	20 min	7
Ghorbani et al. (2014)	Iran	RCT	0/30	15	15	26.06 ± 1.18	26.33 ± 1.30	AE	6	40 min	7
Hemat-Far et al. (2012)	Iran	RCT	0/20	10	10	18–25	18–25	COM	8	40-60 min	3
Hurdiel et al. (2017)	USA	RCT	0/19	10	9	20.1 ± 1.7	20.1 ± 1.7	AE	12	90 min	2
Li K. et al. (2022) and Li R. et al. (2022)	Poland	RCT	197/190	195	192	24 ± 4	23 ± 3	COM	12	45 min	5
Lin Latt et al. (2024)	USA	RCT	0/19	8	11	21 ± 2.32	21 ± 2.32	RT	5	40-60 min	3
Philippot et al. (2022)	Belgium	RCT	3/25	13	15	20.69 ± 1.44	20.93 ± 1.94	HIIT	4	10 min	3
Saltan and Ankaralı (2021)	Turkey	RCT	10/54	29	35	18.82 ± 1.07	19.42 ± 1.38	RT	12	10 min	3
Saltan and Ankaralı (2021)	Turkey	RCT	11/52	28	35	18.85 ± 2.50	19.42 ± 1.378	RT	12	10 min	3
Shavaisi et al. (2024)	Iran	RCT	0/60	30	30	21.27 ± 2.5	21.63 ± 1.73	AE	8	20-30 min	3
Wang et al. (2024)	China	RCT	43/44	47	46	19.18 ± 0.32	19.21 ± 0.17	COM	16	60 min	3
Xiao et al. (2021)	China	RCT	48/17	31	34	19.21 ± 1.02	19.71 ± 1.77	COM	12	90 min	3
Xiao et al. (2021)	China	RCT	47/18	31	34	18.95 ± 0.89	19.71 ± 1.77	COM	12	90 min	3
Zhang et al. (2023)	China	RCT	5/13	9	9	24.20 ± 4.07	22.50 ± 5.95	AE	8	60 min	5
Zhang and Jiang (2023)	China	RCT	0/73	34	39	19.23 ± 0.98	19.16 ± 1.05	COM	12	60 min	3
Abavisani et al. (2019)	Iran	RCT	15/47	31	31	23.77 ± 2.69	23.77 ± 2.69	RT	8	60 min	2
Choi et al. (2018)	Korea	RCT	23/40	33	30	20.8 ± 2.3	21.6 ± 2.91	COM	9	120 min	1
De Vries et al. (2018)	Netherlands	RCT	28/69	49	48	20.86 ± 2.30	20.86 ± 2.30	AE	6	60 min	3
Eather et al. (2019)	Australia	RCT	18/35	27	26	20.23 ± 1.72	20.48 ± 2.01	HIIT	8	8-12 min	3
Ji et al. (2022)	China	RCT	76/57	66	67	21.42 ± 2.4	21.90 ± 2.1	COM	6	45 min	2
Ji et al. (2022)	China	RCT	80/51	64	67	22.31 ± 3.1	21.90 ± 2.1	COM	6	60 min	2
Kim (2014)	Korea	RCT	0/27	12	15	21.0 ± 0.2	21.0 ± 0.3	Yoga	12	60 min	1
Li et al. (2015)	China	RCT	36/170	101	105	20.63 ± 1.03	20.92 ± 1.15	COM	12	60 min	5
Li R. et al. (2022)	China	RCT	0/27	14	13	22.6 ± 2.5	22.5 ± 2.0	RT	8	50 min	2
Paolucci et al. (2018)	Canada	RCT	NR	18	18	21 ± 2	21 ± 2	HIIT	6	20 min	3
Paolucci et al. (2018)	Canada	RCT	NR	19	18	21 ± 2	21 ± 2	AE	6	27.5 min	3
Tomar et al. (2023)	Saudi Arabia	RCT	25/0	16	9	18.62 ± 0.88	18.62 ± 0.88	COM	12	30 min	3
Yiğiter and Hardee (2017)	Turkey	RCT	0/30	30	30	20 ± 1.07	19.94 ± 1.24	AE	8	60 min	3
Barği (2022)	Turkey	RCT	3/28	15	16	20 ± 1.07	19.94 ± 1.24	AE	4	30-60 min	5
Fukui et al. (2021)	Japan	RCT	57/68	39	49	21.6 ± 2.7	21.5 ± 1.4	AE	8	35 min	7

**Table 2:** Overview of research outcome and evaluation tools in included literature for meta-analysis.

Author	Outcome						Evaluation tools/content						Dropout Rate%
	Mental health	Wellbeing	Anxiety	Stress	Depression	sleep disorder	Mental health	Wellbeing	Anxiety	Stress	Depression	sleep disorder	
Antony and Tomar (2019)	√						PWS						0%
Arbinaga et al. (2018)	√	√					RSE	SEES					50%
Aşçı (2003)			√						STAI				0%
Brandão et al. (2024)		√	√	√	√			SHS	DASS-21	DASS-21	DASS-21		19.1%
Castellote-Caballero et al. (2024)		√	√	√				WEMWBS	STAI	PSS			15.3%
Chang et al. (2022)		√	√	√	√			14-item WEMWBS	PHQ-4	PSS-10	PHQ-4		65%
De Vries et al. (2016)				√		√				Utrecht Burnout Scale- Student version		VBBA	19%
Erdoğan et al. (2024)				√						PSS			7%
Falsafi (2016)			√	√	√				HAS	SLSI	BDI		20.24%
Ghorbani et al. (2014)			√		√	√			GHQ-28		GHQ-28	GHQ-28	0%
Hemat-Far et al. (2012)					√						BDI		
Hurdiel et al. (2017)						√						PSQI	48.65%
Li K. et al. (2022) and Li R. et al. (2022)	√		√				PWBS		CAS				0%
Lin Latt et al. (2024)	√		√	√	√	√	WHO-5		PDP	PSS-10	PDP	ISI	64.3%
Philippot et al. (2022)			√	√	√				DASS-21	DASS-21	DASS-21		13.33%
Saltan and Ankaralı (2021)					√	√					BDI	NHP	0%
Saltan and Ankaralı (2021)					√	√					BDI	NHP	0%
Shavaisi et al. (2024)			√	√	√				DASS-21	DASS-21	DASS-21		0%
Wang et al. (2024)			√		√				SCL-90		SCL-90		0%
Xiao et al. (2021)			√	√					SRAS	PSS-14			6.1%
Xiao et al. (2021)			√	√					SRAS	PSS-14			11.4%
Zhang et al. (2023)			√		√				SAS		SDS		0%
Zhang and Jiang (2023)			√		√				SCL-90		SCL-90		6.02%
Abavisani et al. (2019)			√						STAI				0%
Choi et al. (2018)				√						BEPSI			10%
De Vries et al. (2018)		√		√				SIM		SIM			4.04%
Eather et al. (2019)				√						PSS			24.5%
Ji et al. (2022)			√			√			SAS			PSQI	26.8%
Ji et al. (2022)			√			√			SAS			PSQI	26.8%
Kim (2014)				√						LSS			11.1%
Li et al. (2015)				√						CPSS			10.9%
Li R. et al. (2022)			√						SAS				0%
Paolucci et al. (2018)			√	√	√				BAI	PSS	BDI		9.8%
Paolucci et al. (2018)			√	√	√				BAI	PSS	BDI		9.8%
Tomar et al. (2023)		√			√			WHO-5			PHQ-9		0%
Yiğiter and Hardee (2017)					√						BDI		0%
Barği (2022)			√		√				BAI		BDI		0%
Fukui et al. (2021)	√						KPDS						32.3%

NR: Not Reported; PWS: Perceived Wellness Survey; RSE: Rosenberg Self-Esteem Scale; SEES: Subjective Exercise Experiences Scale; STAI: State–trait anxiety inventory; SHS: Subjective Happiness Scale; DASS-21: Depression, Anxiety, and Stress Scale - 21 Items; WEMWBS: Warwick-Edinburgh Mental Wellbeing Scale; PSS: The Perceived Stress Scale; PSS-10:10-item Perceived Stress Scale;14-item WEMWBS: Warwick-Edinburgh Mental Wellbeing Scale - 14 items; PHQ-4: Patient Health Questionnaire-4; VBBA: Sleep quality scale of the VBBA; UBOS-S: Utrecht Burnout Scale - Student version; HAS: The Hamilton Anxiety Scale; BDI: The Beck Depression Inventory; SLSI: The Student-Life Stress Inventory; GHQ-28: General Health Questionnaire-28; PSQI: Pittsburgh Sleep Quality Index; CAS: The coronavirus anxiety scale; PWBS: Psychological wellbeing scale; PDP: Psychological Distress Profile; WHO-5: World Health Organization-Five Wellbeing Index; ISI: Insomnia Severity Index; NHP: Nottingham Health Profile; SCL-90: The self-assessment scale of psychological symptoms symptom Checklist-90; SRAS: Self-Rating Anxiety Scale; SAS: the self-rating anxiety scale; SDS: the self-rating depression scale; BEPSI: Brief Encounter Psychosocial Instrument; SIM: single-item measures; LSS: the Life Stress Scale; CPSS: Chinese Perceived Stress Scale; BAI: Beck Anxiety Inventory; PHQ-9: Patient Health Questionnaire-9; KPDS: Kessler psychological distress scale-6.

### 3.3 Risk of bias assessment

**Figure 2:**
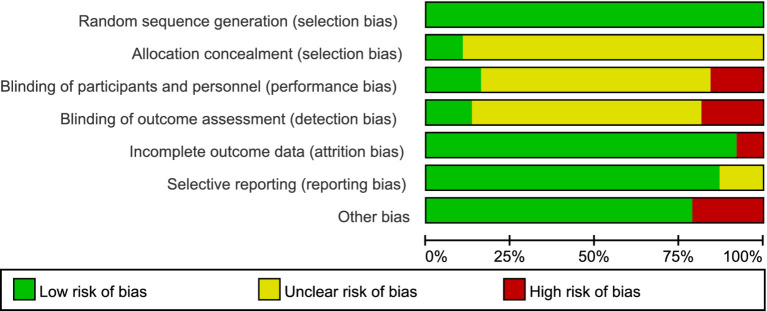
Risk of bias summary. Bar chart summarizing the proportion of studies rated as low, unclear, or high risk across seven domains (random sequence generation; allocation concealment; blinding of participants and personnel; blinding of outcome assessment; incomplete outcome data; selective reporting; other bias). Green = low risk; yellow = unclear risk; red = high risk.

**Figure 3:**
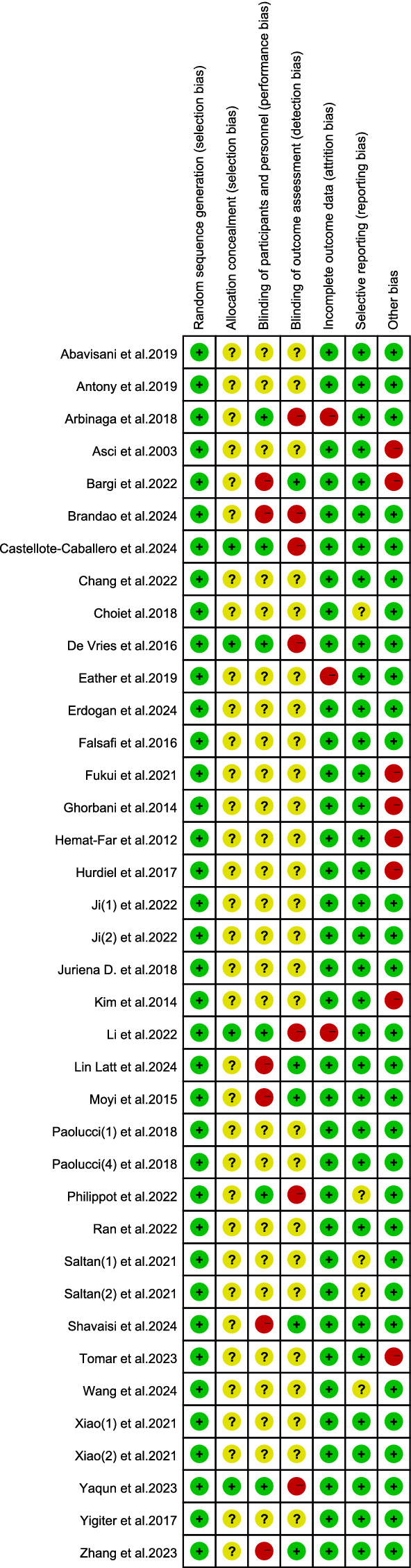
Risk of bias assessments. Traffic-light plot of risk of bias for each included study across the same seven domains. Green = low risk; yellow = unclear risk; red = high risk.Please let us know if anything else is required.

The results of the risk of bias assessment for the included studies are presented in Figures 2, 3. A total of 34 articles clearly reported the method of group allocation, four articles reported allocation concealment, and 10 articles reported blinding of data analysis. In the assessment of data completeness, studies with a dropout rate exceeding 20% and no intention-to-treat analysis were rated as high risk. Among the included studies, 8 had dropout rates over 20%, with comparable dropout numbers and reasons in both the experimental and control groups. Regarding other sources of bias, studies were rated as high risk if they had fewer than 10 participants (small sample bias), lacked supervision of the exercise intervention, or had conflicts of interest. Of the included studies, 30 did not present any other identifiable sources of bias.

The methodological quality assessment diagram of the included studies is shown below.

### 3.4 Results of meta-analysis

#### 3.4.1 Mental health

**Figure 4:**
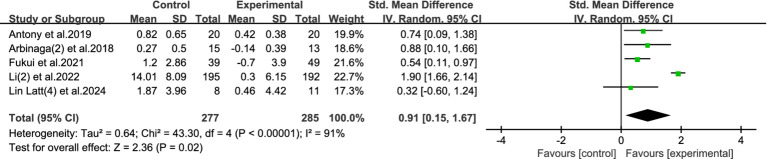
Forest plot of mental health.

In this meta-analysis, we included 5 studies involving a total of 562 participants (277 in the intervention group and 285 in the control group) to assess the effects of physical activity on the overall mental health status of college students. Due to substantial heterogeneity among the included studies (I^2^ = 91%, *p* < 0.00001), a random-effects model was applied. The pooled analysis revealed a significant positive effect of exercise interventions on mental health, with a standardized mean difference (SMD) of 0.91 (95% CI: 0.15 to 1.67, *p* = 0.02), suggesting that physical activity has a moderate to large beneficial impact on the mental health of university students. Refer to Figure 4 for the forest plot representation.

These findings suggest that physical activity is an effective non-pharmacological intervention to enhance the overall psychological wellbeing of college students. The effect size indicates that students who participate in structured exercise programs report notably improved emotional and psychological status compared to those in control groups. Given the barriers to accessing formal psychological services, such as stigma, cost, and limited availability on campuses, regular participation in physical activities offers a practical and accessible alternative for mental health promotion.

Moreover, the significant heterogeneity observed suggests that program duration, intensity, and type of exercise may influence outcomes, emphasizing the need for tailored and sustainable interventions. Future studies could further explore these moderating factors to optimize exercise-based mental health programs in the university setting.

#### 3.4.2 Wellbeing

This meta-analysis included 6 studies involving a total of 1,030 participants (500 in the experimental group and 530 in the control group) to evaluate the effect of physical activity interventions on the subjective wellbeing of university students. Heterogeneity testing showed a very low level of heterogeneity among studies (*I*^2^ = 0%, *p* = 0.73), thus a fixed-effects model was applied for data synthesis.

**Figure 5:**
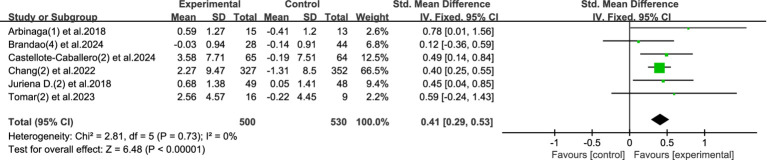
Forest plot of wellbeing.

The pooled results indicated a statistically significant improvement in wellbeing among students who participated in physical activity interventions, with a standardized mean difference (SMD) of 0.41 (95% CI: 0.29 to 0.53, *p* < 0.00001). This represents a moderate effect size, highlighting the positive role of physical activity in enhancing the psychological wellbeing of university students. Refer to Figure 5 for the corresponding forest plot.

The findings support the view that engagement in regular physical activity can meaningfully enhance the sense of wellbeing among college students. A moderate increase in wellbeing is of particular importance for this population, as students often face substantial academic, social, and emotional stressors. Improving wellbeing through accessible, low-cost methods like exercise may serve as a preventive measure against the development of more severe mental health disorders.

Moreover, given the minimal heterogeneity observed, this effect appears to be consistently reproducible across different settings and study designs, which enhances the reliability of the findings and their applicability to campus-wide mental health strategies.

#### 3.4.3 Anxiety

This meta-analysis included 24 studies with a total of 2,466 participants (1,205 in the experimental group and 1,261 in the control group) to investigate the effect of physical activity interventions on anxiety symptoms in university students. The heterogeneity test revealed a high level of heterogeneity among the included studies (*I*^2^ = 82%, *p* < 0.00001), therefore a random-effects model was used for analysis.

**Figure 6:**
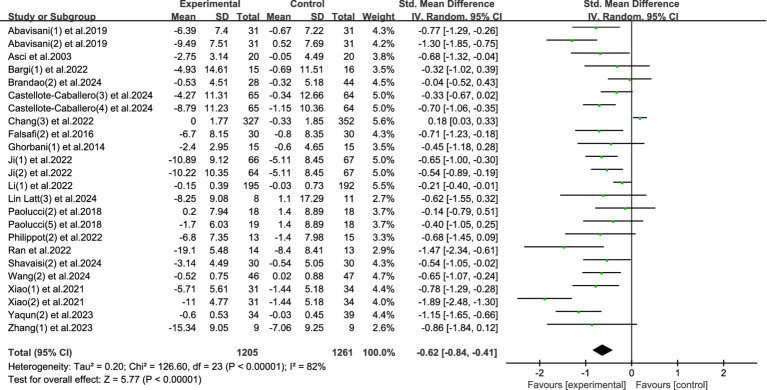
Forest plot of anxiety.

The pooled analysis indicated that physical activity significantly reduced anxiety symptoms, with a standardized mean difference (SMD) of −0.62 (95% CI: −0.84 to −0.41, *p* < 0.00001). This result demonstrates a moderate and statistically significant reduction in anxiety levels in the intervention groups compared to controls. See Figure 6 for the forest plot illustration.

These findings suggest that participation in physical activity is an effective strategy to alleviate anxiety symptoms among university students. Given the increasing prevalence of anxiety in this population, and the limitations and stigma associated with pharmacological or clinical treatments, physical activity provides a non-invasive, accessible, and low-cost alternative. The observed moderate effect size indicates that even relatively short-term or moderate-frequency interventions can yield measurable benefits. Furthermore, the broad inclusion of different exercise modalities (e.g., aerobic, resistance training, mind–body exercises) across the studies enhances the generalizability of the findings.

Despite the high heterogeneity, the consistency in direction and magnitude of effects across most studies reinforces the robustness of the conclusion. Future research should explore the optimal types, intensities, and durations of exercise interventions for anxiety relief, as well as potential gender or cultural moderators.

#### 3.4.4 Stress

This meta-analysis included 19 studies, involving a total of 1,935 participants (941 in the intervention group and 994 in the control group), to evaluate the effects of physical activity on stress levels among university students. The heterogeneity test indicated a moderate level of heterogeneity (*I*^2^ = 67%, *p* < 0.0001), and thus a random-effects model was applied.

**Figure 7:**
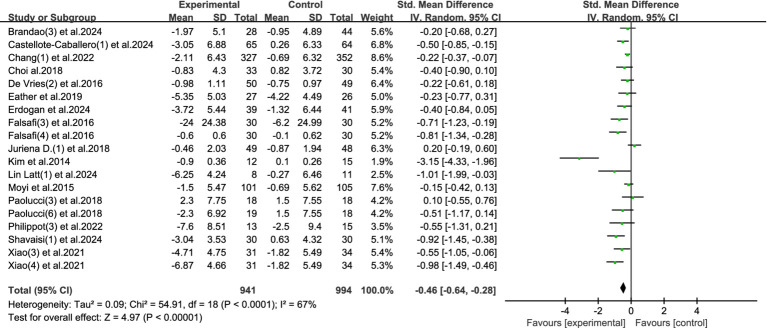
Forest plot of stress.

The pooled results demonstrated a statistically significant reduction in stress in the experimental group compared to the control group, with a standardized mean difference (SMD) of −0.46 (95% CI: −0.64 to −0.28, *p* < 0.00001). This result suggests that physical activity interventions are associated with a moderate improvement in stress levels. Refer to Figure 7 for the forest plot representation.

These findings highlight physical activity as a psychologically empowering and behaviorally accessible method for alleviating stress among university students. Unlike more passive treatment modalities, exercise allows individuals to actively engage in self-regulation, offering a sense of control and agency over their emotional state—factors that are particularly valuable in managing academic and social pressures. Moreover, physical activity not only targets psychological stress but also brings about physiological benefits such as improved sleep quality and autonomic regulation, which may further enhance students’ resilience to stress over time. Its flexibility in form, intensity, and setting enables broad adaptability across diverse student populations and campus environments.

Given the moderate effect size and consistent direction of results, incorporating physical activity into student wellness programs could contribute meaningfully to comprehensive mental health promotion in higher education.

#### 3.4.5 Depression

This meta-analysis synthesized results from 18 studies, comprising a total of 1,469 participants (706 in the experimental group and 763 in the control group), to evaluate the effectiveness of physical activity interventions in alleviating depressive symptoms among university students. Given the substantial heterogeneity across studies (*I*^2^ = 71%, *p* < 0.00001), a random-effects model was applied.

**Figure 8:**
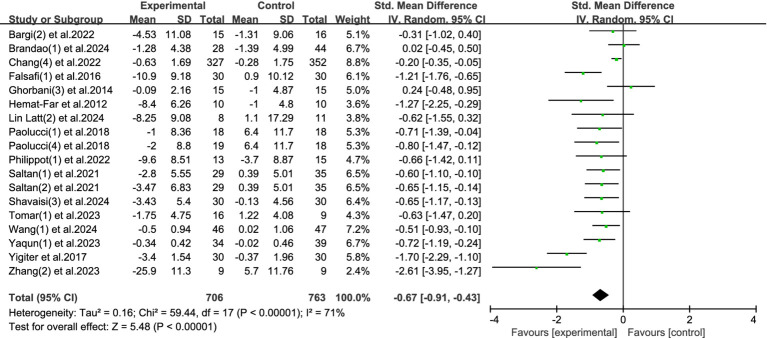
Forest plot of depression.

The combined analysis revealed a significant reduction in depressive symptoms in the exercise groups compared to controls, with a standardized mean difference (SMD) of −0.67 (95% CI: −0.91 to −0.43, *p* < 0.00001). This represents a moderate-to-large effect size, indicating that physical activity has a clinically meaningful impact on improving depression-related outcomes in college populations. Refer to Figure 8 for the visual summary.

The results underscore physical activity as a proactive, self-directed intervention that can help reduce depressive symptoms in young adults. Unlike treatments that rely heavily on clinical infrastructure or pharmacological management, exercise offers a behaviorally autonomous approach—encouraging students to take an active role in managing their emotional wellbeing.

Importantly, the therapeutic benefits of exercise extend beyond mood regulation. It also promotes neurobiological changes (e.g., increased endorphins, improved neuroplasticity) and psychosocial advantages (e.g., increased self-esteem, social connectedness), all of which contribute to its effectiveness in addressing depression.

Although heterogeneity across studies was moderate, the consistent direction and magnitude of results across interventions suggest that physical activity could serve as a core component in campus-based mental health promotion efforts. Further research may clarify optimal program design, but current evidence supports its wide implementation as a preventive and adjunctive strategy for depression in student populations.

#### 3.4.6 Sleep disorder

This meta-analysis included 8 studies, with a total of 558 participants (270 in the experimental group and 288 in the control group), to examine the effects of physical activity interventions on sleep quality among university students. Heterogeneity testing showed no significant heterogeneity (*I*^2^ = 0%, *p* = 0.99), indicating high consistency across studies; thus, a fixed-effects model was used.

**Figure 9:**
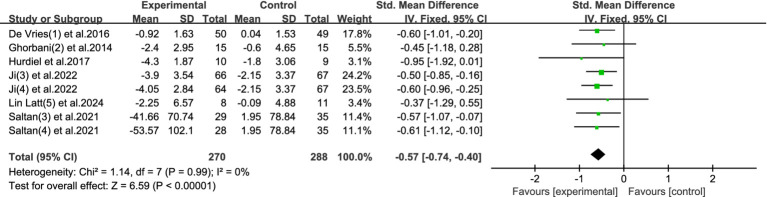
Forest plot of sleep disorder.

The pooled results demonstrated a significant improvement in sleep quality following exercise interventions, with a standardized mean difference (SMD) of −0.57 (95% CI: −0.74 to −0.40, *p* < 0.00001). This indicates a moderate effect size, suggesting that physical activity has a meaningful impact in alleviating sleep-related problems in college students. See Figure 9 for the forest plot representation.

The findings highlight physical activity as a highly consistent and effective intervention for improving sleep quality among university students. Sleep disturbances are increasingly common in this population and are closely linked to poor academic performance, emotional dysregulation, and increased mental health risks. Unlike pharmacological treatments for sleep, which may cause dependency or side effects, exercise offers a physiologically natural approach to restoring sleep patterns by regulating circadian rhythms, reducing arousal, and improving mood. The absence of heterogeneity across studies also reinforces the stability and generalizability of the observed effects.

Given its dual role in promoting both physical recovery and mental restoration, physical activity may serve as an essential part of sleep hygiene strategies in university settings—especially for students experiencing chronic stress or irregular schedules.

### 3.5 Subgroup analysis

#### 3.5.1 Effects of exercise frequency and duration on the mental health of university students

Mental Health: In terms of intervention frequency, studies with fewer than three sessions showed a significant positive effect on psychological wellbeing (SMD = 0.69, 95% CI: 0.25 to 1.13, *p* = 0.002), with no observed heterogeneity (*I*^2^ = 0%). Studies with a frequency of four to fewer than seven sessions also demonstrated a relatively large effect size (SMD = 0.91), but the result was not statistically significant (*p* = 0.070), and high heterogeneity was observed (I^2^ = 96.6%). Regarding intervention duration, studies with a period of 10 to 48 weeks showed a statistically significant and large effect (SMD = 1.36, 95% CI: 0.22 to 2.50, *p* = 0.019), while interventions lasting less than 6 weeks or between 6 and 10 weeks yielded non-significant results. The subgroup with the longest intervention period also showed substantial heterogeneity (*I*^2^ = 91.0%). These results suggest that both low-frequency interventions (≤3 sessions) and longer durations (10–48 weeks) are particularly effective in improving psychological wellbeing among university students. While short interventions may offer quick benefits, longer programs may lead to more lasting effects. In contrast, moderate-frequency or short-duration interventions showed less consistent outcomes, highlighting the need for clearer guidelines and more standardized intervention designs in future research.

Wellbeing: For intervention frequency, both subgroups showed significant effects. Interventions with ≤3 sessions had a larger impact (SMD = 0.51, 95% CI: 0.27 to 0.75, *p* < 0.001) compared to those with 4 to <7 sessions (SMD = 0.35, 95% CI: 0.15 to 0.56, *p* = 0.001), suggesting that lower-frequency interventions may be more effective for improving wellbeing. Regarding intervention duration, 10–48 weeks yielded the most stable and significant result (SMD = 0.51, 95% CI: 0.18 to 0.83, *p* = 0.002). Although the 6–10 week group showed the highest effect size (SMD = 0.78), the wide confidence interval (95% CI: −0.01 to 1.56, *p* = 0.047) suggests uncertainty. Interventions under 6 weeks also showed a moderate and significant effect (SMD = 0.38, 95% CI: 0.24 to 0.52, *p* < 0.001). These findings indicate that lower-frequency interventions (≤3 sessions) may be more effective than moderate-frequency ones in enhancing students’ wellbeing. In terms of duration, longer interventions (10–48 weeks) produced the most reliable results, though short-term programs also showed meaningful benefits. These patterns suggest that both brief, low-frequency sessions and sustained, long-term engagement may be suitable strategies for promoting wellbeing, depending on student needs and program feasibility.

Anxiety: For intervention frequency, interventions with ≤ 3 sessions showed a significant and moderate effect on reducing anxiety (SMD = −0.70, 95% CI: −0.88 to −0.51, *p* < 0.001), with moderate heterogeneity (*I*^2^ = 55.6%). In contrast, interventions conducted 4 to <7 times produced a smaller and non-significant effect (SMD = −0.23, 95% CI: −0.51 to 0.05, *p* = 0.142), with higher heterogeneity (*I*^2^ = 72.3%). Regarding intervention duration, both longer durations (10–48 weeks) and mid-length durations (6–10 weeks) showed statistically significant effects. Specifically, 6–10 weeks had the largest effect (SMD = −0.85, 95% CI: −1.09 to −0.61, *p* < 0.001), while 10–48 weeks also demonstrated a strong effect (SMD = −0.77, 95% CI: −1.14 to −0.40, *p* < 0.001), though with higher heterogeneity (*I*^2^ = 85%). The results suggest that low-frequency interventions (≤3 sessions) are more effective in reducing anxiety than moderate-frequency ones. In terms of duration, 6–10-week programs showed the greatest benefits, followed closely by 10–48-week interventions. These findings support the use of focused short-term interventions or structured mid-term programs to effectively alleviate anxiety symptoms among university students.

Depression: For intervention frequency, interventions with ≤3 sessions demonstrated a strong and significant effect on reducing depressive symptoms (SMD = −0.75, 95% CI: −0.94 to −0.57, *p* < 0.001), with low heterogeneity (*I*^2^ = 15%). In contrast, studies with 4 to <7 sessions showed a smaller, borderline significant effect (SMD = −0.43, 95% CI: −0.86 to 0.01, *p* = 0.053) and higher heterogeneity (*I*^2^ = 76.4%). Regarding intervention duration, all three subgroups demonstrated significant results, with 6–10 weeks producing the strongest effect (SMD = −1.34, 95% CI: −1.88 to −0.81, *p* = 0.000). Interventions of 10–48 weeks also yielded a moderate effect (SMD = −0.67, 95% CI: −0.84 to −0.39, *p* < 0.001), with no observed heterogeneity. The shortest duration group (<6 weeks) showed a smaller effect (SMD = −0.29, *p* = 0.008). These findings suggest that low-frequency interventions (≤3 sessions) are more effective than moderate-frequency ones for reducing depression. In terms of duration, 6–10-week programs produced the most substantial improvements, followed by longer-term interventions (10–48 weeks). These results highlight the potential of targeted mid-length interventions in effectively addressing depressive symptoms among university students.

Stress: For intervention frequency, both subgroups demonstrated significant effects. Interventions with ≤3 sessions showed a moderate effect (SMD = −0.42, 95% CI: −0.72 to −0.13, *p* = 0.04), though with high heterogeneity (*I*^2^ = 75.7%). Interventions delivered 4 to <7 times also had a significant but slightly smaller effect (SMD = −0.33, 95% CI: −0.55 to −0.12, *p* = 0.003), with moderate heterogeneity (*I*^2^ = 49.9%). Regarding intervention duration, all subgroups showed significant stress-reducing effects. The largest effect was observed in the 10–48-week subgroup (SMD = −0.83, 95% CI: −1.38 to −0.28, *p* = 0.003), followed by the 6–10-week group (SMD = −0.57, *p* < 0.001) and the <6-week group (SMD = −0.20, *p* = 0.029). The results indicate that physical activity interventions are effective in reducing stress across various frequencies and durations. Among them, longer interventions (10–48 weeks) produced the most substantial effects, while 4 to < 7 sessions showed slightly better consistency than ≤ 3 sessions. These findings support the application of mid-to-long-term exercise programs, especially when aiming for more stable and pronounced stress relief outcomes.

Sleep Disorder: For intervention frequency, interventions with ≤3 sessions showed a significant and moderate effect (SMD = −0.58, 95% CI: −0.75 to −0.41, *p* < 0.001), with no observed heterogeneity (*I*^2^ = 0%). The single study with 4 to < 7 sessions showed a non-significant result (SMD = −0.45, 95% CI: −1.18 to 0.28, *p* = 0.225). Regarding intervention duration, the <6-week subgroup produced a significant effect (SMD = −0.55, 95% CI: −0.75 to −0.30, *p* < 0.001), while the 10–48-week group also showed a significant but slightly larger effect (SMD = −0.63, 95% CI: −0.91 to −0.30, *p* < 0.001). Both subgroups had no heterogeneity (*I*^2^ = 0%). The results suggest that both low-frequency interventions (≤3 sessions) and longer durations (10–48 weeks) are effective in improving sleep quality, with slightly stronger effects observed in the long-duration subgroup. The absence of heterogeneity in all subgroups reinforces the reliability and consistency of these findings. These results support the flexibility of physical activity as a sleep-enhancing strategy, whether implemented as a brief or sustained intervention (Table 3).

**Table 3:** Subgroup analysis of the effects of physical activity on mental health in college students.

Moderating variable	Stratified subgroup	Number of effect sizes	SMD (95% CI)	*P*-value	I2 (%)	*P*-value for heterogeneity
Mental health						
Intervention frequency	Intervention Frequency ≤3 times	3	0.69(0.25, 1.13)	0.002	0.0%	0.655
	Intervention Frequency 4 times	/	/	/	/	/
	4 times < Frequency ≤7 times	2	0.91(0.16, 1.67)	0.070	96.6%	0.000
	Intervention Duration ≤6 weeks	1	0.32(−0.60, 1.24)	0.497	0.0%	0.000
Intervention duration	Intervention Duration 6 weeks < Duration ≤10 weeks	2	0.62(0.24, 1.00)	0.001	0.0%	0.458
	Intervention Duration 10 weeks < Duration ≤48 weeks	2	1.36(0.22, 2.50)	0.019	91.0%	0.001
Wellbeing						
Intervention frequency	Intervention Frequency ≤3 times	4	0.51(0.27, 0.75)	0.000	0.0%	0.892
	Intervention Frequency 4 times	/	/	/	/	/
	4 times < Frequency ≤7 times	2	0.35(0.15, 0.56)	0.001	17.7%	0.270
	Intervention Duration ≤6 weeks	3	0.38(0.24, 0.52)	0.000	0.0%	0.513
Intervention duration	Intervention Duration 6 weeks < Duration ≤10 weeks	1	0.78(0.01, 1.56)	0.047	0.0%	0.000
	Intervention Duration 10 weeks < Duration ≤48 weeks	2	0.51(0.18, 0.83)	0.002	0.0%	0.827
Anxiety						
Intervention frequency	Intervention Frequency ≤3 times	17	−0.70(−0.88, −0.51)	0.000	55.6%	0.000
	Intervention Frequency 4 times	/	/	/	/	/
	4 times < Frequency ≤7 times	7	−0.23(−0.51, 0.05)	0.142	72.3%	0.106
	Intervention Duration ≤6 weeks	10	−0.33(−0.62, −0.04)	0.026	74.0%	0.000
Intervention duration	Intervention Duration 6 weeks < Duration ≤10 weeks	7	−0.85(−1.09, −0.61)	0.000	9.2%	0.358
	Intervention Duration 10 weeks < Duration ≤48 weeks	7	−0.77(−1.14, −0.40)	0.000	85.0%	0.000
Depression						
Intervention frequency	Intervention Frequency ≤3 times	12	−0.75(−0.94, −0.57)	0.000	15.0%	0.298
	Intervention Frequency 4 times	/	/	/	/	/
	4 times < Frequency ≤7 times	6	−0.43(−0.86, 0.01)	0.053	76.4%	0.001
	Intervention Duration ≤6 weeks	8	−0.29(−0.51, −0.07)	0.008	25.5%	0.225
Intervention duration	Intervention Duration 6 weeks < Duration ≤10 weeks	5	−1.34(−1.88, −0.81)	0.000	64.3%	0.024
	Intervention Duration 10 weeks < Duration ≤48 weeks	5	−0.67(−0.84, −0.39)	0.000	0.0%	0.981
Stress						
Intervention frequency	Intervention Frequency ≤3 times	14	−0.42(−0.72, −0.13)	0.04	75.7%	0.000
	Intervention Frequency 4 times	/	/	/	/	/
	4 times < Frequency ≤7 times	5	−0.33(−0.55, −0.12)	0.003	49.9%	0.092
	Intervention Duration ≤6 weeks	8	−0.20(−0.37, −0.02)	0.029	23.9%	0.239
Intervention duration	Intervention Duration 6 weeks < Duration ≤10 weeks	6	−0.57(−0.78, −0.36)	0.000	3.8%	0.392
	Intervention Duration 10 weeks < Duration ≤48 weeks	5	−0.83(−1.38, −0.28)	0.003	86.2%	0.000
Sleep disorder						
Intervention frequency	Intervention Frequency ≤3 times	7	−0.58(−0.75, −0.41)	0.000	0.0%	0.988
	Intervention Frequency 4 times	/	/	/	/	/
	4 times < Frequency ≤7 times	1	−0.45(−1.18, 0.28)	0.225	0.0%	0.000
	Intervention Duration ≤6 weeks	5	−0.55(−0.75, −0.30)	0.000	0.0%	0.978
Intervention duration	Intervention Duration 6 weeks < Duration ≤10 weeks	/	/	/	/	/
	Intervention Duration 10 weeks < Duration ≤48 weeks	3	−0.63(−0.91, −0.30)	0.000	0.0%	0.782

#### 3.5.2 Effects of different types of physical activity on anxiety, depression, stress, and sleep disorders in university students

**Figure 10:**
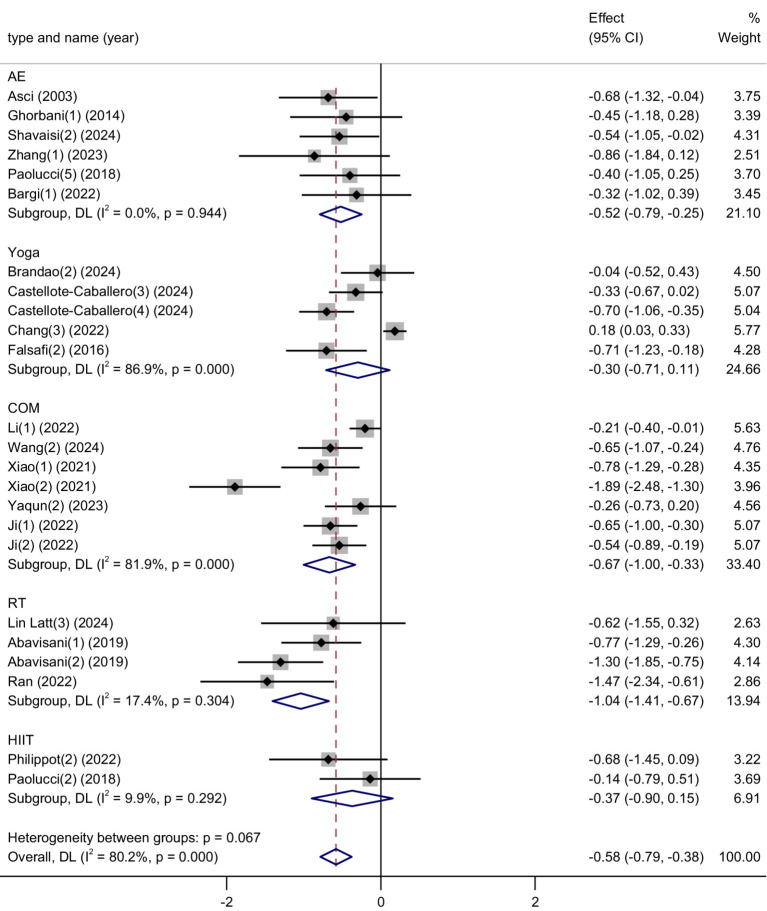
Subgroup analysis of the effects of different exercise types on anxiety symptoms.

Anxiety: Subgroup analysis based on exercise type revealed notable variation in the effects of physical activity on anxiety symptoms. Resistance training (RT) produced the largest reduction in anxiety (SMD = −1.04), followed by combined exercise programs (COM) (SMD = −0.67), both showing considerable effect sizes. Aerobic exercise (AE), yoga, and high-intensity interval training (HIIT) showed relatively smaller effects in comparison. While statistical differences between subgroups were not pronounced (*p* = 0.067), the trend suggests that resistance-based and multimodal interventions may offer greater benefits for anxiety reduction among university students (Figure 10).

**Figure 11:**
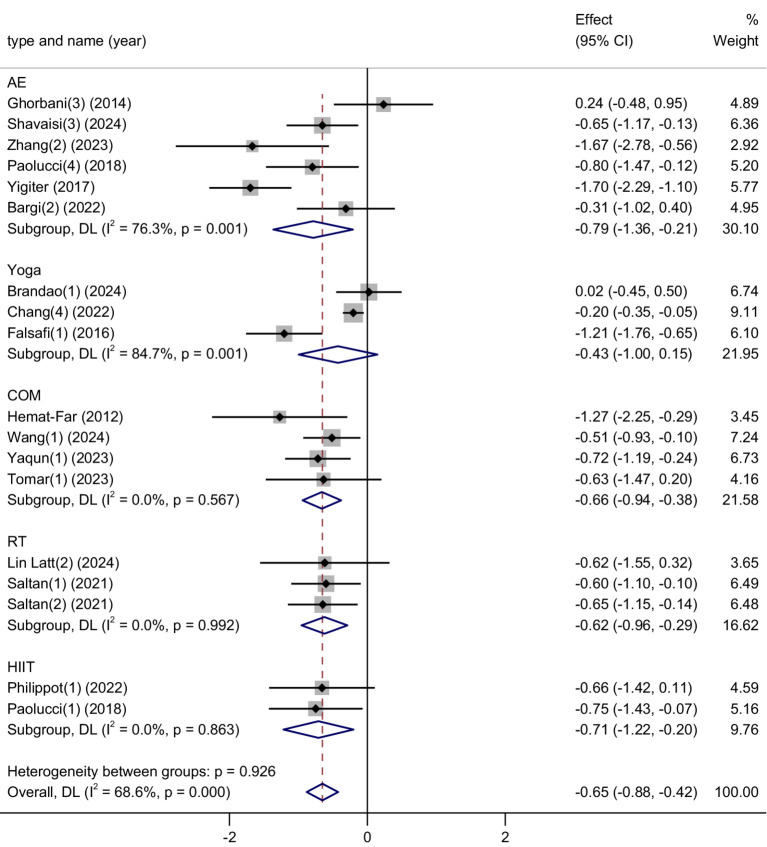
Subgroup analysis of the effects of different exercise types on depression symptoms.

Depression: Subgroup analysis revealed that high-intensity interval training (HIIT) was associated with the greatest reduction in depressive symptoms (SMD = −0.71), indicating it may be the most effective exercise modality for improving depression among university students. Combined exercise (COM) and resistance training (RT) also produced substantial effects (SMDs = −0.68 and −0.65, respectively), suggesting their potential as strong alternatives. In contrast, aerobic exercise (AE) and yoga showed relatively smaller improvements. Although the differences between subgroups were not statistically significant (*p* = 0.926), the trend highlights HIIT as a particularly promising intervention for depression reduction (Figure 11).

**Figure 12:**
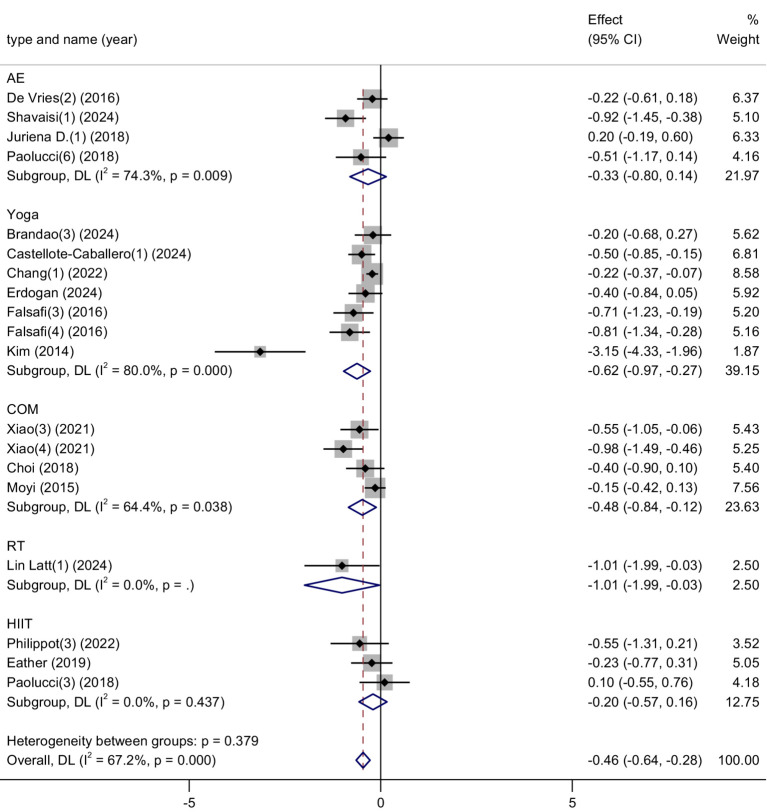
Subgroup analysis of the effects of different exercise types on stress symptoms.

Stress: The subgroup analysis showed that resistance training (RT) had the strongest effect in reducing stress among university students (SMD = −1.01), suggesting it may be the most effective exercise modality for stress relief. Combined exercise (COM) and yoga also demonstrated moderate effects (SMD = −0.65 and −0.40, respectively). Aerobic exercise (AE) and high-intensity interval training (HIIT) showed smaller effects in comparison. Although the test for subgroup differences was not statistically significant (*p* = 0.379), the trend clearly favors RT as a particularly impactful approach for managing stress through physical activity (Figure 12).

**Figure 13:**
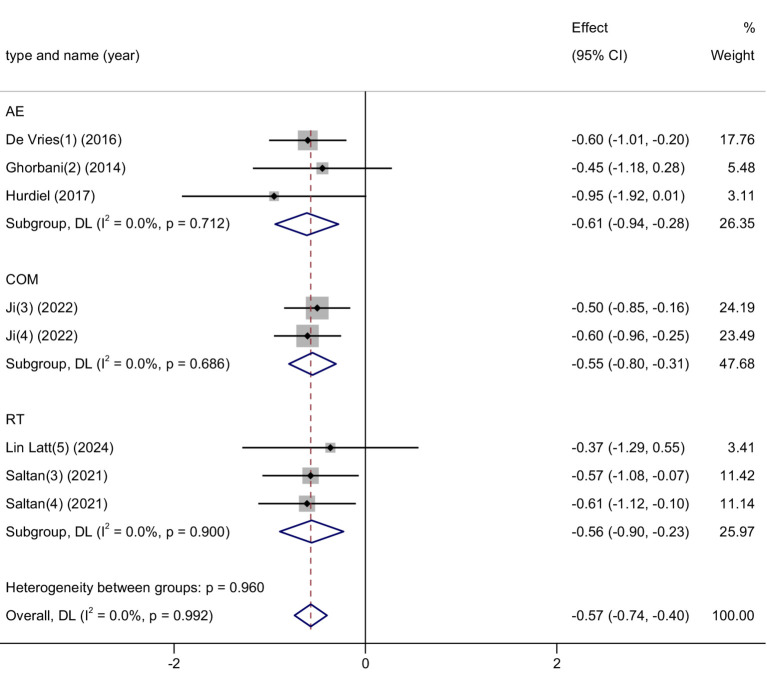
Subgroup analysis of the effects of different exercise types on sleep disorder symptoms.

Sleep Disorder: Subgroup analysis revealed that all three exercise modalities—aerobic exercise (AE), combined exercise (COM), and resistance training (RT)—were effective in improving sleep quality among university students. Among them, aerobic exercise showed the largest effect size (SMD = −0.61), suggesting it may be the most effective option for reducing sleep problems. COM and RT followed closely with similar levels of benefit (SMD = −0.55 and −0.56, respectively). Although differences between subgroups were minimal (*p* = 0.960), the findings point to aerobic exercise as a particularly promising and accessible strategy for improving sleep in this population (Figure 13).

### 3.6 Sensitivity analysis

**Figure 14:**
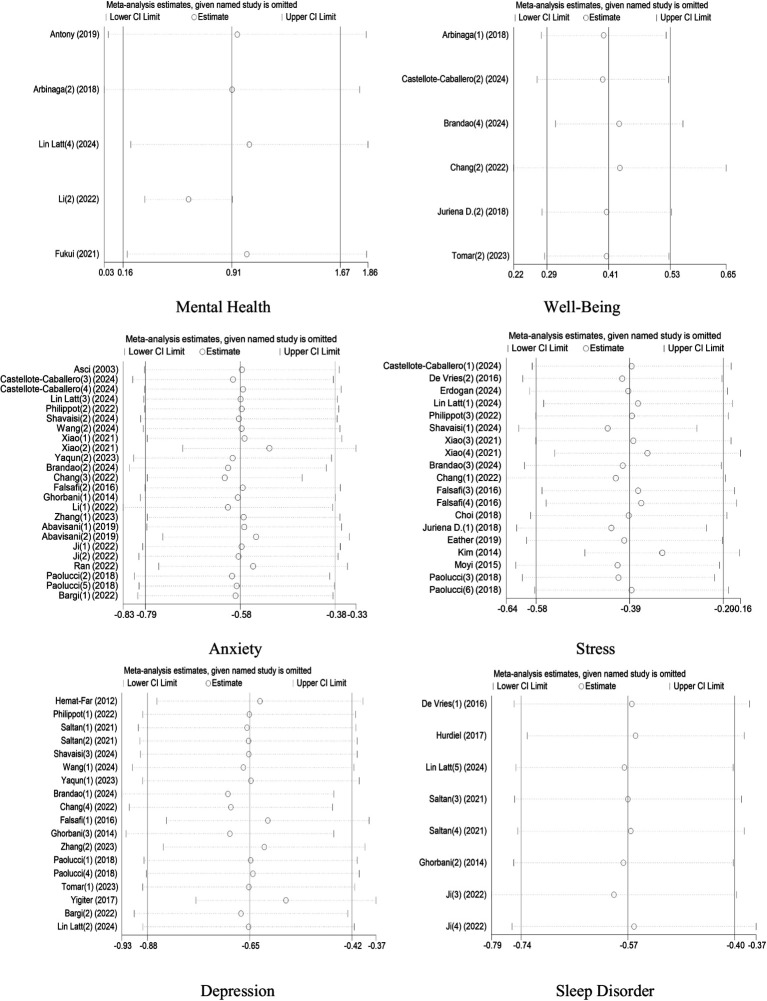
Sensitivity analysis of the effects of physical activity on mental health in college students.

To assess the robustness of the meta-analysis results, a sensitivity analysis was performed using a leave-one-out approach across all six outcome dimensions. The results showed that no single study significantly altered the overall effect size in any domain, indicating that the findings are stable and not overly influenced by any individual study. Across the dimensions of mental health, wellbeing, anxiety, stress, depression, and sleep disorders, the pooled effect sizes remained within a consistent range when each study was removed in turn. This suggests that the meta-analytic results are robust and reliable, and that the conclusions drawn are not dependent on specific studies with extreme results or large weights (Figure 14).

### 3.7 Publication bias analysis

**Figure 15:**
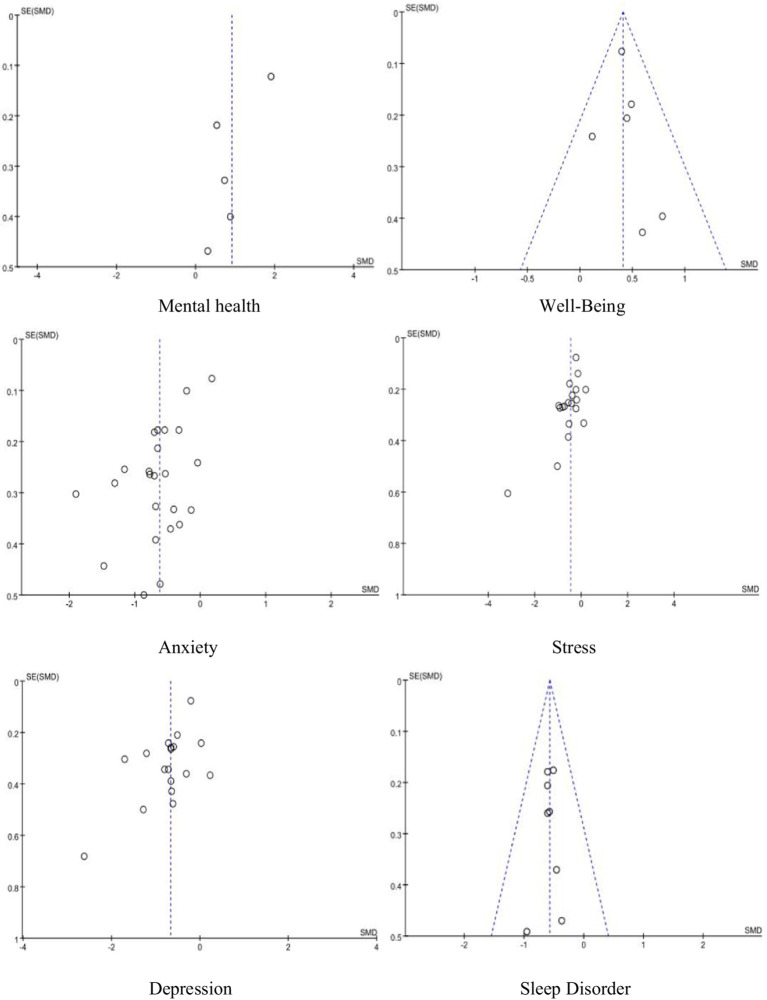
Funnel plot of publication bias.

To reduce the impact of publication bias and verify the robustness of the results, this study employed the Trim-and-Fill method. Through this method, we filled in the missing studies and adjusted for bias. After the adjustment, the effect estimates for anxiety and depression showed minimal changes, and the overall conclusions remained unchanged. This suggests that even after bias correction, the results remain consistent and show no significant variation. Therefore, the adjusted analysis indicates that while there is some bias in the anxiety and depression outcomes, the overall effect remains robust after applying the Trim-and-Fill method. To reduce the impact of publication bias and verify the robustness of the results, the Trim-and-Fill method was applied. This method fills in missing studies that could potentially contribute to publication bias and recalculates the overall effect size. For the anxiety and depression outcomes, which showed significant publication bias (with Egger’s test *p*-values of 0.000 and 0.007, respectively), the Trim-and-Fill method revealed that adjusting for missing studies had only a marginal effect on the overall effect estimates. The adjusted effect sizes for anxiety and depression remained stable and did not reverse the overall conclusions. This suggests that while small-study effects may have inflated the results, the effect sizes for anxiety and depression are still robust even after accounting for potential publication bias (Figure 15).

## 4 Discussion

Over the past two decades, the prevalence of mental health symptoms among university students has nearly doubled (Duffy et al., 2019). A recent multinational study reported that 18.5% of surveyed students met the diagnostic criteria for major depressive disorder, while 16.7% met the criteria for generalized anxiety disorder (GAD) within the preceding 12 months (Auerbach et al., 2018). Consequently, the mental health of university students has emerged as a critical area of concern for public health and national development strategies. This systematic review and meta-analysis evaluated the impact of active participation in physical activity on mental health outcomes among university students. The findings demonstrate that regular engagement in physical activity is significantly associated with improvements in mental health, including the prevention of psychological disorders and the enhancement of overall wellbeing. Additionally, physical activity was found to reduce symptoms of anxiety and stress, contribute to the prevention of depression, and improve sleep quality in this population.

This meta-analysis was conducted to evaluate the effectiveness of physical activity in improving mental health outcomes among university students. The pooled analysis of the included studies demonstrated statistically significant improvements across a range of psychological indicators. Specifically, a standardized mean difference (SMD) of 0.91 was observed for overall mental health (95% CI: 0.15 to 1.67, *p* = 0.02), indicating a moderate-to-large beneficial effect. Wellbeing showed a moderate improvement (SMD = 0.41, 95% CI: 0.29 to 0.53, *p* < 0.00001). Significant reductions were identified for anxiety (SMD = −0.62, 95% CI: −0.84 to −0.41, *p* < 0.00001), stress (SMD = −0.46, 95% CI: −0.64 to −0.28, *p* < 0.00001), and depressive symptoms (SMD = −0.67, 95% CI: −0.91 to −0.43, *p* < 0.00001). Improvements in sleep disorders were also reported, with a moderate effect size (SMD = −0.57, 95% CI: −0.74 to −0.40, *p* < 0.00001). These findings provide empirical support for physical activity as an effective and accessible non-pharmacological intervention for enhancing mental health in university student populations. The results highlight the potential applicability of exercise-based strategies in higher education settings, particularly in light of the structural and psychological barriers that frequently limit access to conventional mental health services. Subgroup analyses revealed that both the frequency and duration of physical activity interventions influenced their effectiveness across mental health outcomes. Interventions conducted three or fewer times per week were consistently associated with statistically significant and moderate-to-large improvements in overall mental health (SMD = 0.69), wellbeing (SMD = 0.51), anxiety (SMD = −0.70), depression (SMD = −0.75), stress (SMD = −0.42), and sleep quality (SMD = −0.58). These results were generally characterized by low or no heterogeneity, indicating high reliability. In contrast, interventions delivered 4 to fewer than 7 times per week tended to yield smaller or non-significant effects and were accompanied by higher heterogeneity, suggesting less consistent outcomes. With respect to intervention duration, programs lasting 10 to 48 weeks produced the most robust and stable effects across all measured outcomes, including overall mental health (SMD = 1.36), wellbeing (SMD = 0.51), anxiety (SMD = −0.77), depression (SMD = −0.67), stress (SMD = −0.83), and sleep quality (SMD = −0.63). Notably, interventions lasting 6 to 10 weeks also demonstrated strong effects, particularly for anxiety (SMD = −0.85) and depression (SMD = −1.34), highlighting the efficacy of mid-length programs. Although shorter interventions (<6 weeks) remained generally effective, their effect sizes were comparatively smaller.

Further subgroup analyses based on exercise modality revealed differential effects across mental health domains. For anxiety, resistance training (RT) was associated with the greatest reduction (SMD = −1.04), followed by combined exercise (COM; SMD = −0.67), while aerobic exercise (AE), yoga, and high-intensity interval training (HIIT) exhibited relatively smaller effects. Although the between-group difference did not reach statistical significance (*p* = 0.067), the trend suggests that resistance-based or multimodal interventions may confer greater benefits for anxiety reduction. Regarding depressive symptoms, HIIT yielded the largest effect size (SMD = −0.71), followed by COM (SMD = −0.68) and RT (SMD = −0.65). AE and yoga were associated with smaller improvements. While the subgroup difference was not statistically significant (*p* = 0.926), HIIT appeared to be a particularly promising modality for the management of depression.

For stress reduction, RT again demonstrated the strongest effect (SMD = −1.01), followed by COM (SMD = −0.65) and yoga (SMD = −0.40), whereas AE and HIIT showed more modest effects. The difference across subgroups was not significant (*p* = 0.379), yet the findings suggest that RT may be particularly effective for alleviating stress. In the context of sleep disorders, all three examined modalities—AE, COM, and RT—were found to be effective. AE produced the largest effect size (SMD = −0.61), slightly surpassing COM (SMD = −0.55) and RT (SMD = −0.56). Subgroup differences were minimal and non-significant (*p* = 0.960), indicating that all modalities are beneficial, with AE potentially serving as the most accessible and impactful option for improving sleep quality.

Overall, these findings indicate that the effectiveness of physical activity interventions may vary depending on both the targeted mental health outcome and the type of exercise employed. Tailoring intervention strategies based on specific symptom profiles and individual preferences may enhance the efficacy of physical activity as a mental health intervention among university students.

The findings of the present study are consistent with those reported in previous meta-analyses (Ruiz-Hernández et al., 2022; Reyes-Molina et al., 2022; Huang et al., 2024). For instance, a systematic review investigating the effects of physical exercise on the mental health of adolescents aged 12–26 years concluded that physical activity exerts positive effects on both anxiety and depression (Pascoe et al., 2020). The current study not only supports these conclusions but also extends the existing evidence by demonstrating that physical activity contributes to improvements in stress-related symptoms and sleep disorders among university students. Furthermore, the results indicate that engaging in physical activity three times or fewer per week is more effective in enhancing mental health outcomes than exercising 4 to fewer than 7 times per week. One possible explanation is that more frequent exercise sessions may lead to psychological fatigue or burnout, thereby attenuating the overall mental health benefits. In addition, longer intervention durations—spanning several weeks—were associated with more pronounced improvements, potentially due to sustained participation facilitating the development of psychological resilience, improved physical health, and enhanced self-efficacy. These findings suggest that low-frequency, long-duration exercise interventions may constitute a particularly effective strategy for promoting mental health in university student populations. Nevertheless, the underlying mechanisms warrant further investigation in future research.

Several studies have identified notable gender differences in university students’ participation in physical activity, with female students often exhibiting higher participation rates than their male counterparts. This observation is consistent with existing literature indicating that female college students are at greater risk of developing mental health disorders compared to males (Sheldon et al., 2021). Women have been found to account for a disproportionately higher share of common psychological conditions, including anxiety and depression (Doherty and Kartalova-O’Doherty, 2010). These findings suggest that female students may be more vulnerable to psychological distress, underscoring the need for gender-sensitive preventive strategies. In particular, external policy interventions—implemented in tandem with efforts to promote participation in extracurricular physical activity—may offer enhanced psychological support for female students and more effectively address the diverse mental health needs of the broader university population.

Existing research has demonstrated that different types of physical activity exert varying effects on mental health outcomes among university students. For example, notable distinctions have been observed between traditional Eastern mind–body exercises and team sports such as basketball or soccer. Xiao et al. (2021) reported that both Baduanjin (a traditional form of Chinese qigong) and basketball interventions were effective in reducing anxiety and stress levels among participants. Moreover, follow-up assessments conducted 2 months post-intervention indicated that these positive mental health effects were sustained over time. These findings reinforce the role of physical activity in promoting psychological wellbeing, particularly in reducing anxiety, feelings of inadequacy, and loneliness. However, the data also suggest that the magnitude of benefit may differ by exercise modality. Group-based basketball interventions appeared to yield greater improvements in mental health outcomes compared to the more solitary Baduanjin practice. One possible explanation is that the social interaction inherent in team sports may contribute more effectively to alleviating psychological distress. Additionally, basketball is typically more intense and competitive than Baduanjin, potentially resulting in increased dopamine release, heightened engagement, and greater enjoyment during participation. These physiological and psychological mechanisms may amplify the mental health benefits of team-based physical activities, underscoring their potential value in university-based mental health promotion strategies.

Mental health status has been shown to vary across age groups, with individuals aged 18 to 24 at the highest risk for developing psychological disorders. As age increases, the prevalence of poor mental health generally declines, rendering the university student population particularly vulnerable. Given the increasing proportion of college students experiencing psychological distress (Lipson et al., 2022), and the positive effects of physical activity interventions identified in the present analysis, the implementation of structured exercise programs may serve as an effective strategy for the prevention and management of mental health issues in this demographic. Good mental health is strongly associated with academic performance. Timely intervention may not only mitigate the long-term impact of psychological disorders on academic achievement (Babaeer et al., 2022), interpersonal relationships, and overall wellbeing (Duffy et al., 2020), but also enhance the likelihood of successful recovery from mental health conditions (Colizzi et al., 2020).

In addition to physical activity, psychological counseling and psychiatric medication are widely recognized as standard approaches for the treatment of mental health conditions. However, research indicates that the majority of students experiencing psychological distress do not seek formal treatment (Eisenberg et al., 2011). Among university students, the utilization rates of counseling services and psychiatric medication remain relatively low. For example, in a study conducted by Blanco et al. (2008), only 16% of students diagnosed with significant anxiety disorders received treatment—a figure that may be partially explained by the inclusion of social anxiety and specific phobias, which typically have lower treatment rates compared to generalized anxiety disorder or panic disorder. Low treatment rates for substance use disorders have been attributed to multiple factors, including perceived stigma (Wu et al., 2003), limited recognition of early symptoms by individuals or their peers, and delayed awareness of the need for care. The lag between the onset of such disorders and the manifestation of serious consequences may also discourage timely help-seeking, particularly among young adults—a demographic in which these behaviors are often normalized, especially within university environments (Wang et al., 2005). In this context, regular participation in physical activity presents a promising complementary or alternative strategy for addressing mental health challenges in university students. Compared to traditional treatment modalities, physical activity offers a less stigmatizing, more accessible, and cost-effective option that can be seamlessly integrated into students’ daily routines. Moreover, it may help alleviate the burden of long wait times commonly associated with access to professional mental health services (Broglia et al., 2018), while also providing enhanced privacy—an important factor influencing students’ willingness to engage in support-seeking behaviors. These advantages highlight the potential of structured exercise interventions as a practical and forward-looking approach to promoting mental health in university populations.

## 5 Limitations

Although this study provides a comprehensive assessment of the impact of physical activity on the mental health of university students through systematic review and meta-analysis, several limitations must be acknowledged when interpreting the results. First, substantial heterogeneity was observed among the included studies, particularly with respect to intervention type, frequency, duration, and assessment tools. This variability may affect the robustness and generalizability of the findings. Future meta-analyses with larger sample sizes may yield more generalizable conclusions and facilitate more detailed subgroup analyses (e.g., by gender, region, or academic stage). Second, this study included only English-language publications, which may have resulted in the exclusion of relevant non-English studies and introduced potential language bias. Third, since all participants in the included studies were undergraduate students, the generalizability of the findings is limited. Future research should thus include more diverse student populations to enhance the external validity of the results. Additionally, although our discussion acknowledged gender differences in mental health risks and participation, the current meta-analysis did not include subgroup analyses based on gender composition, which is a notable limitation for a nuanced interpretation. Furthermore, some of the included studies had small sample sizes, lacked supervision during interventions, or did not report blinding procedures, which may increase the risk of bias. Although sensitivity analyses supported the stability of the results, these methodological limitations may still influence the overall interpretability of the findings.

## 6 Conclusion

From an integrative perspective, research has shown that participation in physical activities lasting at least 4 weeks effectively improves mental health in university students, alleviating symptoms of depression, anxiety, and stress, while enhancing wellbeing and sleep quality. Notably, interventions characterized by lower frequency (≤3 sessions per week) and longer duration (10–48 weeks) appear to yield the most significant benefits. Subgroup analysis on exercise modalities revealed that certain types, such as resistance training and combined exercise, have stronger effects on mental health outcomes compared to aerobic exercises or yoga. To effectively enhance the mental health of university students, universities should develop and promote structured physical activity programs that align with these findings. Specifically, programs could emphasize low-frequency (e.g., ≤3 sessions per week) and longer-duration (e.g., 10–48 weeks) interventions, incorporating exercise modalities such as resistance training or combined exercise given their observed stronger effects on anxiety, depression, and stress. For instance, promoting weekly exercise programs combining 2 resistance training sessions with 1 aerobic session, or encouraging participation in team-based combined exercise activities, could be highly beneficial.

Faculty members should encourage students to engage in physical exercise to reduce academic stress and foster teamwork. Students, in turn, should actively participate in sports to develop self-management skills, cultivate healthy habits, and build resilience, thereby better equipping themselves to cope with academic and life challenges. Emphasizing physical activity is crucial for improving mental health outcomes. Further studies are needed to optimize exercise types, intensity, and duration in order to gain a deeper understanding of their effects on mental health. Future research should also explore potential influencing factors such as student lifestyle, academic pressure, and exercise preferences.

## Data Availability

The datasets presented in this study can be found in online repositories. The names of the repository/repositories and accession number(s) can be found in the article/supplementary material.

## Footnotes

^1^ https://www.crd.york.ac.uk/prospero/
